# Assessing the mediating role of motivation in the relationship between perceived management support and perceived job satisfaction among family doctors in Jiangsu province, China

**DOI:** 10.1186/s12960-023-00849-x

**Published:** 2023-08-28

**Authors:** Xinglong Xu, Lulin Zhou, Sabina Ampon-Wireko, Prince Ewudzie Quansah

**Affiliations:** School of Management, Jiangsu University, Zhenjiang, 212013 People’s Republic of China

**Keywords:** Family doctors, Motivation, Perceived management support, Job satisfaction, Jiangsu province

## Abstract

**Background:**

The study aimed to examine the influence of perceived management support on job satisfaction through the mediating role of motivation among family doctors in the Jiangsu province of China.

**Methods:**

Six dimensions of motivation were employed as mediators in the association between perceived management support and job satisfaction in collecting data to analyze the hypothesized relationships in the present study. A total of 600 questionnaires were distributed to family doctors in Jiangsu province. Structural equation model (SEM) in the analysis of a moment structure (AMOS) version 26 software was used to estimate the path coefficients.

**Results:**

Perceived management support has a significant positive relationship with job satisfaction. Motivation had a fully mediated relationship with the association between perceived management support and job satisfaction.

**Conclusions:**

The study's findings suggest motivation is important in enhancing family doctors’ satisfaction and must not be underestimated. It, therefore, offers diverse recommendations for enhancing motivation among healthcare professionals.

## Introduction

An effective healthcare system requires motivated healthcare workers [1]. Healthcare professionals' undesirable work environment has been a vital determinant that causes health care workers to leave for better prospects elsewhere. Workplace dissatisfaction contributes to burnout, high employee turnover, and major underemployment in healthcare facilities [2]. Work satisfaction affect customer satisfaction and overall service quality [3]. Dissatisfied workers are likely to leave their jobs and the remaining staff can provide low-quality service [4].

This forces some businesses to make significant investments in expensive machinery, capital equipment, and complex technical systems, which typically results in the neglect of the business's most important resource: its workforce [6]. It is important to note that productivity is typically influenced by aspects that are more human-related. Even if one invests in the latest technologies, productivity would barely rise in such a company if staff members waste time and money, are frequently absent, steal, and are not committed to their jobs. As a result, an organization's employees are a necessary ingredient for increased production and achieving the organization's goals and objectives [7].

Mahmoud [5] explores the impact of demographic characteristics on satisfaction among Ethiopian health workers using the logistic regression process. The analysis revealed that management support was strongly linked to the job satisfaction of the five independent variables. SM Assiri, SF Shehata and MM Assiri [6] used structural equation modelling to find a significant correlation between organizational support and employee satisfaction. In industrialized and developing economies, job satisfaction has been a significant measure of health services' quality and success [7–9]. X Li, Y Zhang, D Yan, F Wen and Y Zhang [10] discovered that perceived management support would positively affect job control, resulting in increased job satisfaction among Chinese nurses. According to Y Lu, X-M Hu, X-L Huang, X-D Zhuang, P Guo, L-F Feng, W Hu, L Chen and Y–T Hao [11], high job satisfaction can boost employee morale and enhance an organization’s efficiency and development.

Previous studies have discussed the effects of age and marital status[12, 13], educational level [14], and insufficient training opportunities [15, 16] on job satisfaction among health workers worldwide. Limited (if any) studies have explored the relationship between perceived management support and job satisfaction among family doctors in China. Due to the dearth of studies examining how management support and motivation impact job satisfaction among family physicians, the current study aims to address the following fundamental inquiries: how does perceive managerial support impact job satisfaction? What connection exists between job satisfaction and motivation? What further role does motivation play in mediating the connection between perceived managerial support and job satisfaction?. To fill the gap in the literature, this study has three objectives. First, by considering the organizational support theory of Eisenberger, the study aims to understand better the influence of perceived management support on family doctors’ job satisfaction. Second, we explored the relationship between employees’ motivation and job satisfaction. Finally, the study examines the mediating role of the family doctor’s motivation in the association between management support and job satisfaction. This goal is based on the notion that when family doctors feel the health management team respects their contributions, they are more likely to be inspired, sequentially resulting in job satisfaction. This study is essential, as China's government's interest is in reducing the growing turnover retention among health workers. The study will provide appropriate and specific policy guidelines to achieve high job satisfaction and performance among health workers. This study adds to previous studies on the value of management support for health workers' satisfaction and well-being. Health managers can also use this study to develop plans to boost family doctors' morale in China, allowing them to remain an essential part of the healthcare system.

## Theoretical background and hypotheses

Job satisfaction is an important variable used to increase employees' productivity in the workplace [17]. The study depicts ideas drawn from various sources of literature and self-determination theory, and the social exchanged theory that have been incorporated into this theoretical framework. This is due to the complexities of job satisfaction and the fact that there is no ready-made solution or single answer to what makes people happy at work. As a result, the relationship between motivation and job satisfaction has recently received much empirical attention in the healthcare industry. Although the precise understanding of motivation is still evolving [21] most theories have classified motivation as either extrinsic or intrinsic and then investigated its effects on job satisfaction [23]. According to those theories, extrinsically motivated behaviours are governed by an external mechanism (e.g., incentives or punishment), whereas intrinsically motivated behaviours are directed by personal interest [22]. However, extant literature has suggested that individuals’ behaviour can sometimes not be well-explained by either intrinsic interest or extrinsic incentives [23]. For instance, employees may engage in work activities, because they feel responsible for their work or because they identify with the importance of the work rather than, because they feel interested in their work (i.e., intrinsically motivated) of pressured to do it (external regulation). This sense of identifying the work as important is also a type of motivation within self-determination theory (SDT) and could, therefore, also promote various work outcomes [24]. SDT was built on the classic distinction between extrinsic and intrinsic motivation and presented a continuum model of controlled versus autonomous [25]. According to the continuum model, extrinsic motivation can be differentiated into multiple regulation types: extrinsic regulation, introjected regulation and identified regulation [25]. This multidimensional conceptualization of motivation and thus provides a more nuanced way to explore the relationship between motivation and job satisfaction.

**Fig. 1:**
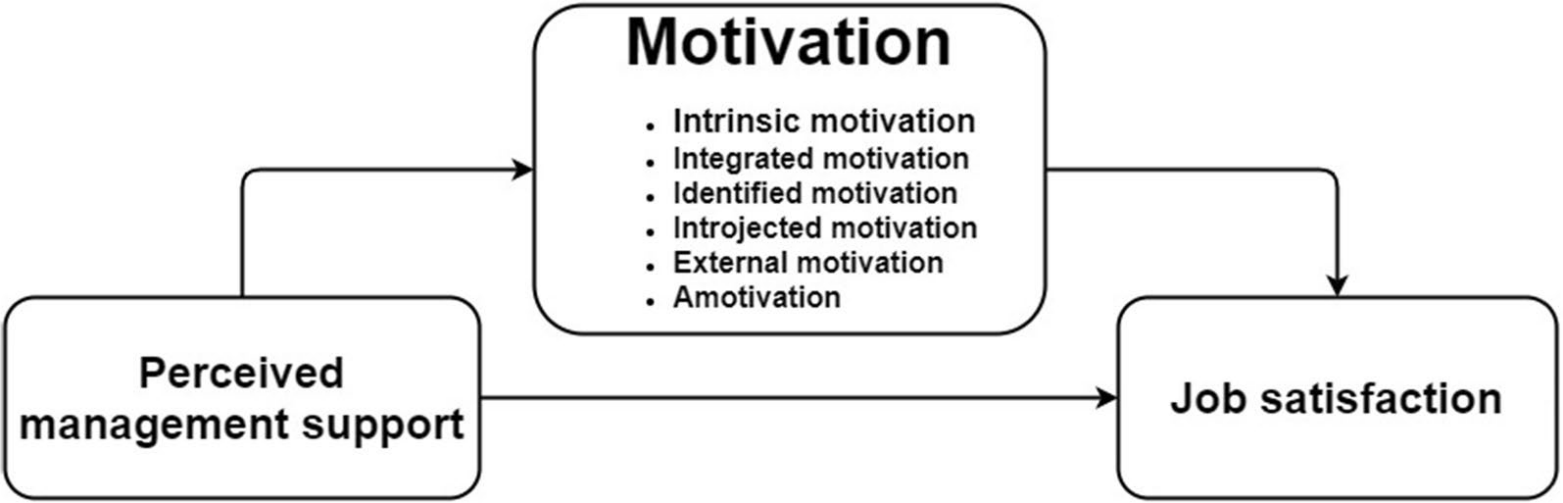
Conceptual framework of the study

Our first contribution is to advance previous research by assessing perceived management support and job satisfaction. Furthermore, the mediating effects of the detailed motivation types of motivation in the relationship between perceived management support and job satisfaction among family doctors. Figure 1 depicts the theoretical framework that has been established.

## Perceived management support and job satisfaction

Perceived management support (PMS) refers to employees' perceptions of how much an organization values their contributions and how well they are handled [18]. Management support can be in compensation, career enrichment, rewards, promotions, and verbal recognition [19]. The Social exchange theory [20] and AW Gouldner [21] are the foundations of management support. According to the social exchange theory, when an employee’s conditions are exact, the individual who has been owed a favour will return the favour [20]. Moreover, employees see their employment as a conduit for a cooperative partnership between management and staff that exposes relative dependence and goes beyond the formal contract. Workers who are assisted by their superiors feel secure and have a good assertiveness toward the organization with some sort of belonging. In contrast, employees who do not receive assistance can exhibit emotional behaviors, leading to exhaustion, frustration, and alienation, depending on the circumstances [22].

A significant positive relationship between management support and job satisfaction has been established. According to a study by M Armstrong‐Stassen [23], employers with high PMS levels have higher job satisfaction than those without. This outcome could be defined as PMS enhancing individuals' confidence and opinions about their employers' values and rewards [24]. According to research, PMS initiates a social exchange mechanism through which individuals feel obligated to help the company achieve its objectives, resulting in higher rewards. As a result, employees are more comfortable with their work and reciprocate organizational support in various ways. Employees with PMS experience may have an inherent obligation to be socio-emotionally committed to their job and the company. It is, therefore, proposed that:

H1: perceived management support will have a significant positive influence on job satisfaction among family doctors.

## Motivation and job satisfaction

Another critical factor influencing job satisfaction among health professionals is motivation. Motivation is a term that describes an external state that encourages a particular behaviour and demonstrates that behaviour in internal responses [25].

Quality promotion of healthcare services has become one of the most challenging goals of healthcare systems all over the world [26–28]. Research findings regarding the influence of motivation and satisfaction have been inconclusive. For instance, a study in Turkey showed no statistical difference in overall satisfaction and overall motivation ratings [29]. Another study emphasized that just because healthcare professionals are happy with their jobs does not mean they will be inspired as well [30]. On the contrary self-motivation and job satisfaction were found to be positively related [31]. However, K Toode, P Routasalo and T Suominen [32] revealed that motivation is a crucial predictor of healthcare professionals' responses to challenges and stresses. According to Bonenberger [33], motivation and job satisfaction appear to be highly linked to satisfaction. It is, therefore, reasonable to hypothesize that,

H2: motivation (H2-a-intrinsic; H2-b-integrated; H2c-identified; H2-d-introjected; H2e-external; H2f-amotivation) will significantly affect job satisfaction among family doctors.

## Management support and motivation

Employees’ confidence in their employer to consider their efforts, well-being, and meet their social and emotional needs is measured by perceived management support [34, 35]. Management support theory emphasis that workers are more likely to shape positive responses to organizations if they feel the company supports them in their efforts [19]. R Miao and H-G Kim [36] study in Taiwan evaluated the association between organizational support and employee motivation, and the results revealed a strong connection between the two variables. Employee morale and administrative support are inextricably linked and essential for any successful organization. Employee motivation and organizational support have a good relationship, according to the findings [37]. K Imtiaz, M Farooq, MS Hashm and R ul Aain [38] considered the relationship amid employee engagement and perceived corporate support in Pakistan and came up with similar results. A study in Pakistan found that management support positively correlated with motivation. Grounded on the above literature, the study hypothesizes that:

H3: perceived management support will have a significant influence on motivation (H3-a-intrinsic; Hb-b-integrated; H3c-identified; H3-d-introjected; H3e-external; H3f-amotivation).

## Mediation role of motivation

Some studies have established the extent to which management support could significantly and directly influence motivation[39, 40] and job satisfaction [41, 42]. Other studies have also indicated that motivation significantly influences job satisfaction in the workplace. Perceived management support helps employees develop positive psychological attitudes that compel them to be motivated about their work [43]. Motivated employees may show greater satisfaction with their job, because their organization helps them meet their needs. An increased employee motivation leads to a higher level of job satisfaction [44]. Motivated and satisfied employees assume that they are an integral part of the organization, can achieve their objectives, have a sense of control over their work, and participate in activities that support the organization [45, 46].

Notwithstanding the evidence on how these three constructs are directly related to each other in different literature, finding a study that combines these constructs in a single model will be more worthy. This phenomenon is supported by the fact that individuals who gain management support feel motivated about their work, and the long-run effect may be manifested in job satisfaction [47, 48]. Therefore, examining motivation as a mediator in perceived management support and job satisfaction relationship will address the existing gap in the literature. On this note, we hypothesize that.

H4: motivation (H4-a-intrinsic; H4-b-integrated; H4c-identified; H4-d-introjected; H4e-external; H4f-amotivation) will mediate the connection between perceived management support and family doctors job satisfaction.

## Methods

### Research design

This was a quantitative study with a cross-sectional observational design. The manuscript was written per the Strengthening reporting of Observational Studies in Epidemiology (STROBE) protocol [49].

### Participants and procedure

According to the National Bureau of Statistics in China [50], Jiangsu province has approximately 1028 community hospitals known as health service centers. Each community hospital has approximately 50 to 100 beds and 15 doctors. The hospital offers basic medical and healthcare services within the small towns in the district. The present study focused on Southern, Central, and Northern Jiangsu community hospitals. According to the National Bureau of Statistics of China, this is similar to the layout of Eastern China, Central China, and Western China. Community hospitals were selected, because their function has recently emerged more important in China during the new medical reform [51].

Since there was a vigorous lockdown due to the COVID-19 outbreak at the time of the study, most hospitals were designated as epicentres for the treatment of COVID-19 disease and fever-related diseases. For this reason, the researchers purposively contacted the departmental heads of 410 community hospitals for their consent to proceed with the study. Surprisingly, only three hundred and thirty-three 333 community hospital heads responded affirmatively to allow their facilities to be used for the study. Participants were recruited between 12th February 2020 till 11th September 2020. All enrolled participants were informed of the study’s purpose and procedures and provided written informed consent. After receiving permission to gather the data, the researchers went to the hospitals and gave the family doctors invitation packages. The package contained a plain-language report and a consent form questionnaire in Chinese. It took each participant approximately 25 min to complete the survey. We assured the participants that their involvement was voluntary, with anonymity guaranteed. The researchers further gave respondents a prepaid envelope. This enabled them to send the filled survey to the corresponding author. The researchers issued the prepaid envelopes to assure the participants of optimum confidentiality. Approximately 600 questionnaires were distributed to participants.

Descriptive analysis was performed to show the demographic characteristics of the participants. Out of the 600 questionnaires, 486 were returned, showing a response rate of 81%. 240 (58.1%) respondents were females, and 173 (41.9%) were males. The average age of the participants was 29, with a standard deviation of 4.67. The participants' average worked for 4.69 years, recording a standard deviation of 0.89. The married participants were 315 (76.3%), and the unmarried participants were 98 (23.7%).

### Measures

All items were measured on a 7-point Likert scale. This study's instruments were adapted from existing scales with reliability and validity established. Because the leaves were used in China, we converted the rankings into Chinese to ensure the translated scales' consistency and validity following recommended practices [52]. Two language translator experts who are organisational behaviour professors and have received accredited bilingual translation certificates did the translation. Using these experts was necessary as they also assessed the content strength of the scales. The process involved translation and back-translation, correcting antiquated languages, and using nouns rather than pronouns. The questionnaire also considered the views of others (e.g., insiders' and outsiders’ perspectives). The translation was vital, because it provided the researchers with information and source-language transparency on validity [53]. Finally, we piloted the Chinese questionnaires on two different samples to be convinced about the converted scales’ reliability and strength.

The first and second samples included 169 and 201 physicians excluded from the final study. In the first and second samples, perceived management support had a reliability Cronbach alpha of 0.88 and 0.82, respectively. The combined Cronbach alpha for the motivation scale in the first and second samples was 0.73 and 0.77, respectively. The Cronbach alpha for perceived job satisfaction in the first and second samples was 0.80 and 0.91. The test–retest survey results show that the translated scales had acceptable internal consistency and were reliable and valid for use.

### Perceived management support (PMS)

To measure perceived management, we relied on eight items from the PMS Survey [19]. The scale has received significant recognition from previous studies, such as PB Le and H Lei [54], LJ Labrague and JAA De los Santos [55], PS Thompson, DM Bergeron and MC Bolino [56]. The reliability coefficient (Cronbach alpha) of SPOS originally was 0.93, with item-total correlations from 0.42 to 0.83. The PMS's items for our study were slightly modified to suit the current study’s objective without affecting the scale’s conceptual meaning. For instance, the sample item “Our company cares about employees’ well-being” was changed to “This hospital cares about my well-being”. In addition, the sample item “Our firm strongly considers employees’ goals and values” was changed to “This hospital strongly considers the objectives and values of physicians. The Cronbach alpha of the PMS in the current study is 0.905.

### Motivation

The motivation construct was measured with eighteen items from the Work Extrinsic and Intrinsic Motivation Scale (WEIMS) [57]. The eighteen items assess six motivation dimensions, including intrinsic motivation (3 items), introjected motivation (3 items), integrated motivation (3 items), external regulation (3 items), identified motivation (3 items), and amotivation (3 items). The WEIMS is proven to have high internal reliability in previous studies V Gupta [58]. Each of the items was a response to the question “What do you or would involve yourself in your present work?” along a 7-point Likert scale from 1 (does not correspond at all) to 7 (correspond exactly). Examples of the items include “Because I gain much pleasure from learning new things” (intrinsic motivation; Cronbach alpha in the first, second and third samples were 0.80, 0.77 and 0.87, respectively), “Since it has become an integral part of my identity.” (integrated motivation; Cronbach alpha in the first, second and third samples were 0.83, 0.84 and 0.80, respectively), “As it is the style of work I have decided to accomplish a number of important goals” (identified motivation; Cronbach alphas in first, second and third samples were 0.67, 0.74 and 0.70, respectively), “Because I want to be a “winner” in life” (introjected motivation: Cronbach alphas in the first, second and third samples were 0.70, 0.71 and 0.76, respectively), “For the income it provides me” (external regulation; Cronbach alphas in the first, second and third samples were 0.77, 0.81 and 0.73, respectively), and “I don't know, I guess we're supposed to do so much” (amotivation; Cronbach alphas in the first, second and third samples were 0.64, 0.60 and 0.75, respectively) and their Cronbach alphas as presented from the original scale were reliable. In this current study, the reliability coefficients (Cronbach alphas) for intrinsic, integrated, identified, introjected, external and amotivation are 0.873, 0.945, 0.935, 0.816, 0.851 and 0.849, respectively.

### Perceived job satisfaction (JS)

The perceived job satisfaction was assessed with four items from the study of [59] and JR Hackman and GR Oldham [60]. These items had high reliability in previous studies. For instance, HSud Khan, M Zhiqiang, AM Sadick and A-AI Musah [61] recorded 0.891 as Cronbach alpha and 0.592 as the average variance extracted for the four items. Sample items include “I am mostly happy with the work I do.,” “My job is interesting,” “My job is often dull and monotonous”, and “My job is satisfying.” In this current study, the Cronbach alpha for the perceived job satisfaction construct is 0.803.

### Control variables

Variables such as gender, age, salary, work experience, and marital status affect employee job satisfaction [62]. However, we declined to control them in our model, since they were not significantly related to this study's job satisfaction variable.

### Common method bias test

We employed various steps to handle common method variance in our data. While designing and distributing the questionnaires, we followed the proposed steps of PM Podsakoff, SB MacKenzie, J-Y Lee and NP Podsakoff [63]. The steps included randomizing the items' order, and issuing reports to the respondents that the research was solely for academic purposes. In addition, we informed the respondents that they should feel free to choose any answer they deemed fit, and that there was no right or wrong answer. Furthermore, participants are more motivated to be more accurate if they believe the information provided will benefit them or the organization, and promising feedback may also motivate greater accuracy. For this reason, we assured the respondents that the information they provided would enable the design of specific policy guidelines to encourage management support, increase specific motivation and achieve high job satisfaction.

Again, we kept the survey items short to minimize redundant measures and overlaps that helped the participants to give more accurate responses. Respondents were assured that their responses would remain anonymous to alleviate assessment concerns and social desires. We further employed Harman’s one-factor test to detect threats of common method bias. An unrotated, principal component factor examination of all measurement items showed eight factors with eigenvalues above one. The first factor explained 26.85% of the total variance, which is less than 50%, while all elements explained 74.04 per cent of the total variance.

### Data analysis

An essential component of the research study is the appropriate methodological choice, according to Davis (1996) and Stevens (2002). The study employed a second-generation multivariate structural equation modelling approach to assess the relationship between the study variables. The SEM, unlike the other statistical methods assisted in determining validity and reliability of the model metrics. Preliminary analysis was performed using SPSS v. 26.0 and the analysis of a moment structures (AMOS) Version 26 was used for testing the hypothesized relationships. The study used a two-stage technique [64, 65] to estimate the hypotheses. First, we conducted a confirmatory factor analysis (CFA) to assess the variables’ unidimensionality, validity, and reliability. During this process, we employed series tests to compare a theoretical measurement model of the study variables. Second, we specified the hypothesis to examine the fit of the structural model. The study estimated the path coefficients for statistical significance and overall model fit assessments.

## Results

### Confirmatory factor analysis (CFA), reliability and validity analysis

Table 1 shows the summarized findings of the first step. The CFA factor loadings for all the measures were above the suggested 0.50 thresholds (ranging from 0.696 to 0.99) except for one item (JS4) from the job satisfaction scale, which recorded a factor loading of 0.455. According to N Malhotra and S Dash [66], if a measurement item is less than the suggested factor loading threshold and does not affect the reliability or validity of the particular scale, such an item must be retained for further analysis. In this current study, JS4 did not affect the reliability and validity values of the job satisfaction scale; hence, we included it for subsequent analysis.

**Table 1:** CFA loadings and internal reliability testing

Variables	Item code	Estimate	S.E	*t* value	P	C–α	CR	AVE
Integrated motivation (Integ)	Integ1	0.901				0.945	0.949	0.862
	Integ2	0.899	0.035	29.133	***			
	Integ3	0.982	0.031	35.556	***			
External motivation (Ext)	Ext1	0.748				0.851	0.861	0.678
	Ext2	0.956	0.063	17.432	***			
	Ext3	0.748	0.065	15.49	***			
Identified motivation (Ident)	Ident1	0.865				0.935	0.937	0.832
	Ident2	0.876	0.041	25.123	***			
	Ident3	0.99	0.039	30.367	***			
Intrinsic motivation (IM)	IM1	0.957				0.873	0.877	0.706
	IM2	0.778	0.043	18.647	***			
	IM3	0.773	0.043	18.489	***			
Job satisfaction (JS)	JS1	0.793				0.803	0.812	0.53
	JS2	0.783	0.062	15.86	***			
	JS3	0.819	0.063	16.475	***			
	JS4	0.455	0.065	8.826	***			
Introjected motivation (Introj)	Introj1	0.771				0.816	0.818	0.599
	Introj2	0.765	0.069	13.958	***			
	Introj3	0.786	0.065	14.143	***			
Amotivation (Amo)	Amo1	0.71				0.849	0.854	0.665
	Amo2	0.958	0.08	16.248	***			
	Amo3	0.758	0.074	14.822	***			
Perceived management support (PMS)	PMS1	0.696				0.905	0.905	0.545
	PMS2	0.745	0.081	13.999	***			
	PMS3	0.78	0.081	14.599	***			
	PMS4	0.769	0.078	14.418	***			
	PMS5	0.724	0.076	13.622	***			
	PMS6	0.749	0.077	14.07	***			
	PMS7	0.699	0.074	13.188	***			
	PMS8	0.738	0.079	13.865	***			

*C–α* Cronbach’s alpha, *CR* Construct reliability, *AVE* Average variance extracted, *CR* Construct reliability, *AVE* average variance extracted

***p < 0.001

The study employed Cronbach’s alpha to explore the scale’s internal reliability. The scales’ reliability coefficients are between 0.803 to 0.945, and they were greater than the 0.70 thresholds suggested by J Nunnally [67], indicating sufficient internal consistency. Regarding the convergent validity, KG Joreskog and D Sorbom [68] and RB Kline [69] have suggested that it could be adequate if the measure’s construct reliability exceeds 0.70 and the average variance extracted (AVE) is above 0.50. The construct reliability coefficients in this current study ranged from 0.812 to 0.949, and the AVE values ranged from 0.53 to 0.862, suggesting acceptable convergent validity for the measures.

The study also employed fit-statistics suggested by LT Hu and PM Bentler [70] to establish the suitability of the data sets. The fit indexes indicated the model had an acceptable fit to the data set with a Chi-square (*χ*^2^) = 487.041, relative Chi-square (*χ*^2^/df) = 1.292, standardized root mean square residual (SRMR) = 0.034, comparative fit index (CFI) = 0.985, Tucker–Lewis fit index (TLI) = 0.983, and root mean square error of approximation (RMSEA) = 0.048.

The study further assessed the AVE's discriminant validity (square root) with Amos Plugin developed by [71]. The discriminant validity values are accessible along the diagonal lines of the latent variable correlation coefficients in Table 2, which suggest sufficient discriminant validity.

**Table 2:** Discriminant validity analysis

	Integ	Ext	Ident	IM	JS	Introj	Amo	PMS
Integ	**0.928**							
Ext	0.226***	**0.823**						
Ident	0.345***	0.207***	**0.912**					
IM	0.04	0.249***	0.048	**0.84**				
JS	0.360***	0.469***	0.409***	0.245***	**0.728**			
Introj	0.184**	0.374***	0.183**	0.260***	0.367***	**0.774**		
Amo	− 0.377***	− 0.330***	− 0.408***	− 0.226***	− 0.393***	− 0.270***	**0.816**	
PMS	0.326***	0.282***	0.320***	0.131*	0.356***	0.340***	− 0.376***	**0.738**

*Note*. Discriminant validity values are presented in bold along with the inter-factor correlation matrix

*Integ* Integrated motivation, *Ext* External motivation, *Ident* Identified motivation, *IM* Intrinsic motivation, *JS* Job satisfaction, *Introj* Introjected motivation, *Amo* Amotivation, *PMS* Perceived management support

*p < 0.05, **p < 0.01, *p* < 0.001

*** means significant at 95% confidence level

### Means, standard deviation, and correlation analysis

Table 3 presents the means, standard deviation, and correlation analysis of the variables under study. The correlation analysis offers some initial support for the hypothesized relationships. It showed that perceived management support correlated with job satisfaction and all six motivation dimensions, suggesting initial support for H1 and H2. All six motivation variables also significantly correlated with job satisfaction, offering some initial support for H3.

**Table 3:** Means, standard deviation, and correlation

	1	2	3	4	5	6	7	8
1. PMS	1							
1. PJS	0.298**	1						
2. IM	0.100*	0.209**	1					
3. Integ	0.283**	0.288**	0.019	1				
4. Ident	0.287**	0.348**	0.037	0.305**	1			
5. Introj	0.297**	0.285**	0.225**	0.157**	0.153**	1		
6. Ext	0.255**	0.367**	0.217**	0.198**	0.190**	0.315**	1	
8. Amo	− 0.327**	− 0.315**	− 0.156**	− 0.323**	− 0.334**	− 0.225**	− 0.249**	1
Means	4.03	3.76	4.3	3.9	3.89	4.45	4.7	3.9
Std.D	1.29	1.36	1.59	1.54	1.46	1.32	1.51	1.4

*PMS* perceive management support, *JS* job satisfaction, *IM* intrinsic, *Integ* integrated, *Ident* identified, *Intoj* introjected, *Ext* External, *Amo* amotivation, *Wexp* work experience

*p < 0.05; **p < 0.01

### Hypotheses testing

**Fig. 2:**
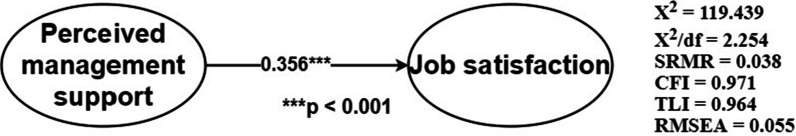
Results of the direct effect of perceived management support on job satisfaction

We tested the hypotheses by examining the connection between perceived management support and job satisfaction (H1). This was done by testing the structural model in Amos version 26 software (Fig. 2). The model gave a good fit to the data (*χ*^2^ = 119.439, *χ*^2^/df = 2.254, SRMR = 0.038, CFI = 0.971, TLI = 0.964, RMSEA = 0.055). The standardised coefficient path from perceived management support to job satisfaction was 0.356, and it is significant (*p* < 0.001). Therefore, it suggests support for H1.

**Fig. 3:**
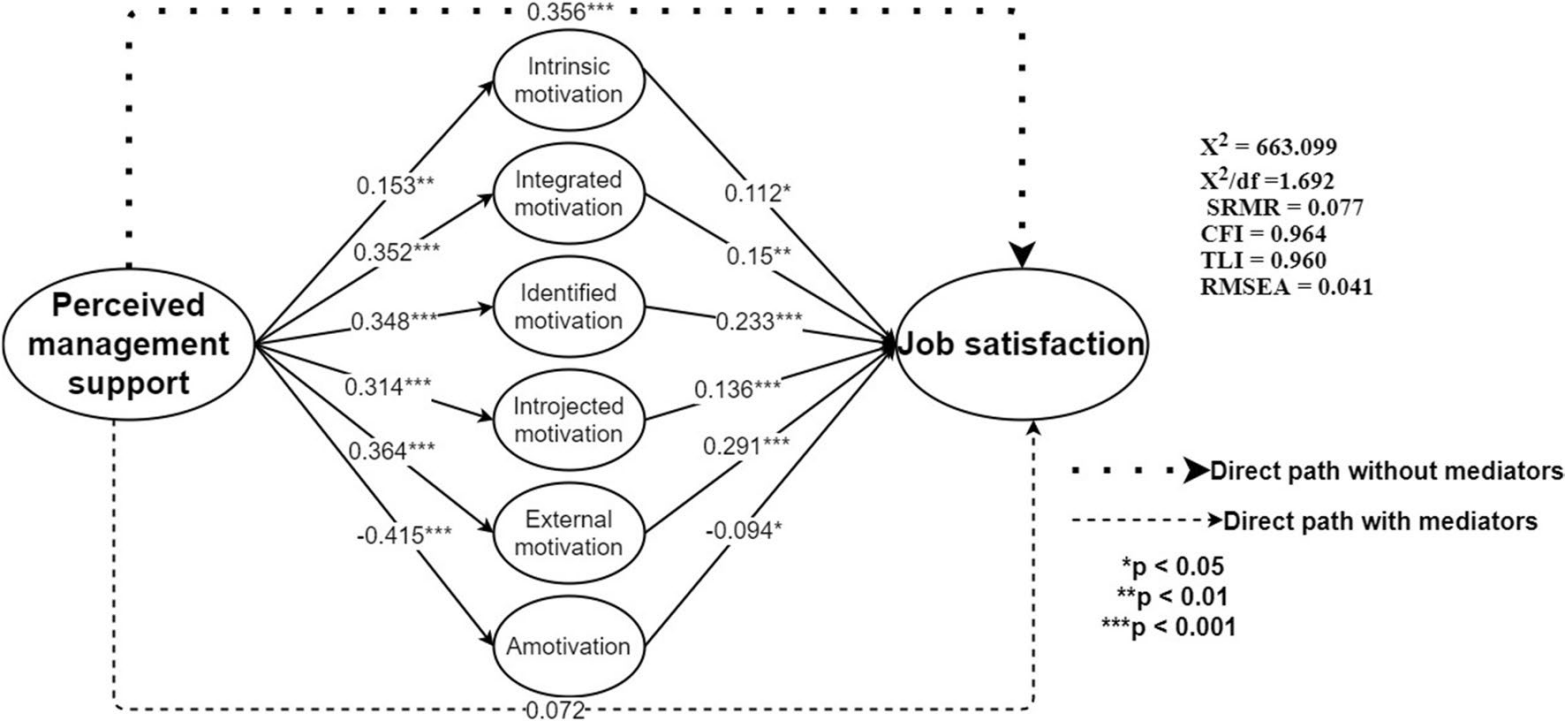
Results of the full structural mediation model showing the mediating effect of six motivation variables in the relationship between perceived management support and job satisfaction

We further made use of ‘Indirect effects, AMOS Plugin tool’ for estimating mediation effect, since the structural equation model (SEM) in AMOS does not directly generate indirect effects estimates. We further tested the full structural mediation model, in which the six components of motivation (intrinsic, integrated, identified, introjected, external, and amotivation) were included in the main effect model (Fig. 3). Rather than using a single mean score to represent the higher order construct-like motivation, we employed intrinsic, integrated, identified, introjected motivation, external and amotivation as first-order indicators of motivation. The approach also helps to identify the contributing mediating role of each motivation dimension. As shown in Fig. 3 the full structural mediation model had a good fit to the data. The model fit (*χ*^2^ = 663.099, *χ*^2^/df = 1.692, SRMR = 0.077, CFI = 0.964, TLI = 0.960, RMSEA = 0.041) indices did not diverge much from the main effect model. The standardized path coefficients from perceived management support to intrinsic, integrated, identified, introjected motivation, external and amotivation were 0.153 (*p* < 0.01), 0.352 (*p* < 0.001), 0.348 (*p* < 0.001), 0.314 (*p* < 0.364), and –0.415 (*p* < 0.001), respectively, confirming H2. About the relationships between each of intrinsic motivation, integrated motivation, identified motivation, introjected motivation, external motivation and amotivation, standard path coefficients were 0.072, 0.112 (*p* < 0.01), 0.15 (*p* < 0.01), 0.233 (*p* < 0.001), 0.136 (*p* < 0.001), 0.291 (*p* < 0.001), and -0.94 (*p* < 0.05), respectively, supporting H3.

In Table 3 is the results of the direct, indirect and total effects from perceived management support and job satisfaction. The standardized indirect effect from perceived management support to job satisfaction through the mediating role of intrinsic motivation was 0.109 with a 5000-sample bias-corrected bootstrapping that generated a 95% confidence interval (CI) of [0.017, 0.167]. According to the rule of thumb, if zero falls within the confidence interval, no significant relationship has occurred. On the contrary, a significant relationship has occurred if zero does not fall within the 95% confidence interval. Based on this, intrinsic motivation mediated the relationship between perceived management support and job satisfaction, hence support H4a. The standardized indirect path from perceived management support to job satisfaction through the mediating roles of integrated motivation (*β* = 0.15, *p* < 0.05; CI = 0.020, 0.182), identified motivation (*β* = 0.233, *p* < 0.001; CI = 0.101, 0.272), introjected motivation (*β* = 0.136, *p* < 0.05; CI = 0.010, 0.199), external motivation (*β* = 0.291, *p* < 0.001; CI = 0.105, 0.269) and amotivation (*β* = − 0.094, *p* < 0.5; CI = − 0.181, − 0.007), did not have zero within their 95% confidence intervals. The results, therefore, support H4b, H4c, H4d, H4e and H4f (see Table 4).

**Table 4:** Direct, indirect, and total effects analysis

Predictors	Job satisfaction	IM	Integ	Ident	Introj	Ext	Amo
Direct effects							
Perceived management support (PMS^a^)	0.356***	–	–	–	–	–	–
PMS^b^	0.072	0.153**	0.352***	0.348***	0.314***	0.364***	− 0.415***
Intrinsic motivation (IM)	0.112*	–	–	–	–	–	–
Integrated motivation (Integ)	0.15**	–	–	–	–	–	–
Identified motivation (Ident)	0.233***	–	–	–	–	–	–
Introjected motivation (Introj)	0.136***	–	–	–	–	–	–
External motivation (Ext)	0.291***	–	–	–	–	–	–
Amotivation (Amo)	− 0.094*	–	–	–	–	–	–
Indirect effects of PMS	95% Confidence interval (CI)						
Through Intrinsic motivation	0.017* (0.019, 0.167)						
Through Integrated motivation	0.053** (0.020, 0.182)						
Through Identified motivation	0.081*** (0.101, 0.272)						
Through Introjected motivation	0.043** (0.010, 0.199)						
Through External motivation	0.106*** (0.105, 0.269)						
Through Amotivation	− 0.039* (− 0.181, − 0.007)						
Total effects of PMS	0.333***						

PMS^a^ refers to the direct effect of perceived management support on job satisfaction from Fig. 1

PMS^b^ refers to the direct effect of perceived management support on job satisfaction from Fig. 2

*p < 0.05; **p < 0.01; ***p < 0.001

## Discussion

Improving workers’ performance has gained attention, especially in the public sector [72]. To the best of our understanding, a handful of investigations have been conducted by previous studies to assess the role of perceived management support and motivational factors in family doctors’ contract services. This is worth exploring for research and policy purposes. The current study examines the impact of management support on family doctors' job satisfaction. The second goal focused on assessing the impact of motivation in mediating the relationship between management support and job satisfaction in Jiangsu province. The results showed that the model's overall structure was appropriate based on fitness indicators' values.

The findings predicted that management support positively impacted job satisfaction, supporting hypothesis 1. This confirms the findings of other scholars [73, 74], who found that workers would give their all to an organization that shows concern for their well-being. The finding implies that when the health management team supports family doctors, they could feel valued, increasing their confidence and trust that the health institution could reward their efforts to attain higher performance [27]. Employees will consequently give back to the health institution in several ways, potentially increasing their level of job satisfaction [29]. The argument is consistent with social exchange theory, which holds that when workers believe their employer values their labor and is concerned about their well-being, they are more likely to feel compelled to engage in actions that are advantageous to the company. The study has consequences for Chinese healthcare administrators and leaders. Health authorities and policymakers can organize family doctors’ jobs, so that self-motivated employees can convey their desire for autonomy and competitiveness.

The study further examined the mediating role of motivation in the relationship between perceived and job satisfaction among family doctors. By so doing, the six components of motivation (intrinsic, integrated, identified, interjected, external, and amotivation) according to the Work Extrinsic and Intrinsic Motivation Scale (WEIMS) [57] were employed. Interestingly, a significant relationship was revealed. Specifically, intrinsic, integrated, identified, introjected, and external motivation consecutively significantly affected the relationship between perceived management support and job satisfaction. Amotivation, on the contrary, had a negative mediatory impact on the relationship between management support and job satisfaction. This implies that, except amotivation, all forms of motivation positively affect the pathway between management support and job satisfaction. A longitudinal investigation should be taken into consideration as it will demonstrate the efficacy of a proposed model between the sample data and predictor variables. The trends of change and the strength of the causal relationship between the targeted variables may be explained in detail using this strategy.

This indicates that when family doctors perceive that their superiors support them, they could be intrinsically motivated and willing to put in a lot of physical and emotional effort for the common good of the hospital. In the case of integrative motivation, the health management team's assistance could increase family doctors’ desire by creating greater internal cohesion and team spirit. Having superiors’ backing would increase their loyalty, dedication, attachment and reduce their desire to leave. The findings support the conclusions of earlier studies which encouraged the need to increase motivation [75, 76]. The implication is that health management can enable family doctors to believe their task is worthwhile. Other specific theories of motivation such as orientation to learning should be taken into account, because they have been widely acknowledged as crucial links between management support and health employee outcomes.

The study results show that PMS could not directly influence job satisfaction when the motivation variables were introduced to create the structural mediation effect model. Since the effects of PMS on job satisfaction completely pass through the types of motivation, the implication is that a full mediation effect has occurred. Interestingly, external motivation had the highest coefficients, and the findings suggest that common rewards such as pay increments, bonuses, promotions, and other benefits stimulate job satisfaction among family doctors. The significant positive effects between perceived management support and motivation are in line with Gillet, Huart, Colombat, and Fouquereau [79, 80], but contradict the findings of Hu and Chang [81]. Therefore, the healthcare management team along with other sectional heads, should implement external motivating elements that would raise employee job satisfaction in the healthcare sector. This is true, because contented employees are productive employees who want to stick around and contribute to the organization’s success.

Introjected motivation also played an essential mediating role in the relationship between management support and job satisfaction. This infers that when the health management team gives their support, it increases family doctors’ aspiration to put in extra effort to avoid guilt and shame and sequentially enhances satisfaction. This agrees with a previous study by A Assor, M Vansteenkiste and A Kaplan [77]. Therefore, health leaders are encouraged to support family doctors to reduce the lack of enthusiasm for engaging in an activity. Another study by T Lam, T Baum and R Pine [78] indicates that management support leads to how satisfied and fulfilled employees are grounded in their motivation.

Besides, the results show that intrinsic motivation improves the pathway through which management support affects work satisfaction. This result is in line with C-K Lee, Y Reisinger, MJ Kim and S-M Yoon [79]. They concluded that voluntary organizational support affects satisfaction, suggesting that increasing employees' intrinsic motivation would encourage them to promote job satisfaction among family doctors. R Imran, K Allil and AB Mahmoud [80] affirmed that intrinsic motivation and imposed rules are essential factors in promoting employee engagement. Furthermore, they discovered that intrinsic motivation has a positive influence on job satisfaction. Moreover, OM Karatepe and M Tekinkus [81] shows that high intrinsic motivation levels are associated with high work efficiency, job satisfaction, and affective loyalty to the organization. Extrinsically, rewards in bonuses, increased salary, or promotion similarly enhanced the relationship between work engagement and job satisfaction among family doctors, and this finding is corroborated by MY Raza, MW Akhtar, M Husnain and MS Akhtar [82].

Unlike the others, amotivation showed a negative relationship with job satisfaction among family doctors in China’s Jiangsu province. The implication is that less motivated doctors are likely to lose interest and enthusiasm for work. Extrinsic motivation revealed a positive relationship with family doctors’ job satisfaction. This indicates that providing external rewards can generate energy that can induce family doctors to be satisfied and aim toward achieving organizational objectives. In another way, motivation is required, because human nature requires encouragement or reward to be satisfied.

## Conclusion

Job satisfaction among healthcare professionals has been a growing concern recently. However, no such model has been explicitly proposed for family doctors in China. Considering family doctors' importance, the study scrutinizes the effects of perceived management support and job satisfaction. Similarly, the impact of perceived management support and motivation is explored. We considered the link between family doctors' motivation and job satisfaction once more. Finally, the attempt to develop motivation as a mediator in the relationship between perceived management support and job satisfaction among family doctors resulted in other results. A total of 600 questionnaires were distributed to the participants. Confirmatory factor analysis (CFA) was used to assess variables' unidimensionality, validity, and reliability. After that, structural equation simulation was used to estimate the hypotheses. As a result, it was discovered that there was a positive association between perceived management support and work satisfaction and all other motivational factors. Motivation, in turn, is a mediator in the relationship between perceived management support and family physicians’ job satisfaction.

## Implications of the study

The current study has several theoretical and practical implications. The study's theoretical implication stems from demonstrating the extent to which perceived management support (PMS) significantly influences family doctors’ job satisfaction (JS). The study did not only investigate the direct relationship between PMS and JS, but went a step further to establish the mediating capacities of six dimensions of motivation (intrinsic, integrated, identified, introjected external, and amotivation) in PMS-JS relationships. Consequently, the study has contributed to the literature by demonstrating that external motivation has the highest predictive capacity in explaining the influence of perceived management support on job satisfaction.

Among the practical implications of the study is demonstrating that employees become satisfied with what they do at work if they receive support from their management. The support the management gives to the family–doctors must seek to address their (family–doctors) needs. This is because different employees may have different needs; hence, addressing someone’s problem may not precisely solve another one’s problem. In addition, management must focus on enhancing the different motivational factors differently, since they explain the impact of management support on job satisfaction differently. A special focus must be given to extrinsic motivation due to its high predictive capacity in the PMS-JS relationship.

## Limitation

The study's findings are interesting but cannot be applied to the entire family of doctors in Jiangsu province, because the sample was drawn from only 33 community hospitals. Therefore, future studies should consider a larger population of family doctors from other provinces of China. This is necessary to ascertain the overall state of health care in China and to develop the necessary attitudes and behaviors for work.

## Data Availability

The data for this research is held by the authors and will be made available upon reasonable request.
